# A new mathematical approach to improve the original dietary inflammatory index (DII) calculation

**DOI:** 10.1371/journal.pone.0259629

**Published:** 2021-11-08

**Authors:** Xenia Pawlow, Raffael Ott, Christiane Winkler, Anette-G. Ziegler, Sandra Hummel

**Affiliations:** 1 Institute of Diabetes Research, Helmholtz Zentrum München, and Forschergruppe Diabetes, Klinikum rechts der Isar, Technische Universität München, Neuherberg, Germany; 2 Forschergruppe Diabetes e.V., Neuherberg, Germany; Istituto di Ricerche Farmacologiche Mario Negri, ITALY

## Abstract

Accumulating evidence links dietary intake to inflammatory processes involved in non-communicable disease (NCD) development. The dietary inflammatory index (DII) designed by Shivappa et al. has been shown to capture the inflammatory potential of dietary behavior in a large number of epidemiological studies. Thus, the DII may serve as future tool to assess someone’s nutritional inflammatory capacities and hence, the individual risks for NCD development later in life. The calculation method of the DII, however, can benefit from alternative mathematical steps, particularly regarding the transformation from standardized daily food consumption to percentile scores. Here, we provide novel approaches, the scaling-formula (SF) and scaling-formula with outlier detection (SFOD) methods, with the aim to optimize the DII calculation method proposed by Shivappa and colleagues. We illustrate on simulated data specific limitations of the original DII calculation and show the benefits of the SF/SFOD by using simulated data and data from the prospective TEENDIAB study cohort, which supports the application of SF/SFOD in future epidemiological and clinical studies.

## Introduction

The prevalence of non-communicable diseases (NCD) is increasing rapidly worldwide [1], accompanied by NCD-caused mortality. Hence, an estimate of around two-third of global deaths in 2017 has been attributed to NCD [2]. Although decreasing NCD death rates have been observed between 2007 and 2017 [2], great efforts are continuously needed to control and reduce NCD numbers, especially in low- and middle-income countries [3], as highlighted by the global action plan of the World Health Organization [4].

A healthy and balanced diet has been implicated as an important lifestyle factor for the prevention of NCD [5]. For example, a Westernized diet, typically rich in (animal-derived) fats and refined carbohydrates and, in parallel, poor in fiber, has been associated with an increased risk for NCD, such as cardio-vascular disease, metabolic syndrome, type 2 diabetes and certain cancers [6,7]. In contrast, subjects consuming a Mediterranean diet, originally rich in plant-based fats, whole grains, fruits and vegetables, may be more protected against certain NCD [8,9]. One plausible explanation for this observation is the anti-inflammatory potential of a Mediterranean diet [10]. Chronic (low-grade) pro-inflammatory processes have been suggested to be causal for a number of NCD, for example, by promoting endothelial dysfunction, a significant contributor to coronary heart disease [11], or by promoting insulin resistance, a major cause for type 2 diabetes [12]. Potentially, such events could therefore be prevented by a higher consumption of foods with anti-inflammatory components, such as fiber [13], vitamin E [14] and n-3 fatty acids [15], and in parallel, lower intake of foods with pro-inflammatory components, e.g., saturated fatty acids [16]. In that sense, an easy-to-use tool to define the inflammatory potential of an individual diet appears to be beneficial to identify and reduce pro-inflammatory food items and promote nutrition with anti-inflammatory effects.

In 2014, Shivappa et al. developed the dietary inflammatory index (DII) using literature-derived information on 45 food parameters and their relation to six inflammatory blood markers, namely interleukin (IL)-1β, IL-4, IL-6, IL-10, tumor-necrosis-factor alpha (TNF)-α and C-reactive protein [17]. Based on the relations between the DII and these biomarkers, there has been a very large number of studies investigating associations between the DII and potential inflammatory-driven diseases. Remarkably, the DII has been applied in over 200 human studies, including different ethnicities and various health outcomes [18–24]. In numerous studies, associations were found between the intake of a rather pro-inflammatory diet (positive DII score) and an increased risk for certain NCD, e.g., several cancer types, cardiovascular disease or type 2 diabetes [18,25–29]. Thus, the DII has the potential to be an easy-to-use and low-cost tool, which could be globally applied, to assess someone’s ‘unhealthy’ diet and potentially prevent NCD development by guiding to a more non-/anti-inflammatory eating behavior. However, not all studies showed consistent associations between the DII and inflammatory markers or health outcomes, for example, with regard to the metabolic syndrome [30]. Moreover, associations in some studies were rather weak [31,32], or were only found in one of the sexes investigated [33–35]. Furthermore, it appears worthy to note that although the original DII is based on the associations between food parameters/nutrients and six selected inflammatory cytokines, several studies did not observe an association between the DII and those cytokines [32,35–39]. One potential reason for these observations could be that the calculation of the DII is still not precise enough to provide a clearer picture in various settings. With the attempt to further improve the DII, we noticed, by mathematically evaluating each calculation step, that the application of the standard normal distribution function in one of those steps does not optimally fit in this context, and that an alternative approach possibly provides a more accurate DII score. Thus, we propose a revised mathematical calculation of the DII, which may result in a higher potential to reveal associations with inflammatory markers and health outcomes, and therefore, may be better suitable for future epidemiological and clinical studies. Furthermore, we describe a possible harmonization approach to compare DIIs across various studies.

## Original DII calculation according to Shivappa et al.

The literature-derived, population-based *DII* calculation by Shivappa et al. has been described elsewhere [17]. For the suggested improvements of the *DII* calculation, the most important assumptions and steps of Shivappa’s *DII* are described briefly. First, fixed relationships are assumed between the considered inflammatory markers (pro-inflammatory: IL-1β, IL-6, TNF-α, C-reactive protein; anti-inflammatory: IL-4, IL-10) and the *DII*. Second, the used articles containing results on the influence of food parameters on these inflammatory markers are selected for the calculation of effect scores of the chosen inflammatory markers. Third, global values (means and standard deviations) for the considered food parameters are estimated from daily consumption data of a global database based on 11 different countries. Furthermore, *P* is assumed as the set of food parameters (a total of 45 food parameters are used by Shivappa et al. [17]) included in the *DII* calculation.

### Scoring algorithm for food parameter effects

For the scoring of selected articles with information on the influence of the 45 food parameters on the six pro-/anti-inflammatory blood markers, let *x* be the result of an article and *a*:*X*→{−1,0,1} be defined through

$$a(x)=\left\{ \begin{array}{c} -1,x=food\;parameter\;showed\;anti–inflammatory\;effect\\ 0,x=food\;parameter\;showed\;no\;inflammatory\;effect\\ 1,x=food\;parameter\;showed\;pro–inflammatory\;effect \end{array}\right.$$

### Raw and overall inflammatory effect scores for a single food parameter

For a fixed food parameter *p*, the scores *a(x*_*pi*_*)* of the selected articles *x*_*pi*_, *i* = 1,…,*n* for the calculation of the effect scores are weighted with the weights *w(x*_*pi*_*)*, depending on study characteristics (study type, study design), e.g. study type *human* and study design *prospective cohort* (for more information see Table 1 in [17]). With it the raw inflammatory effect score (RIES) is calculated by

$${RIES}_{p}=\frac{\sum_{i=1}^{n}a(x_{pi})\times w(x_{pi})}{\sum_{i=1}^{n}w(x_{pi})}\in [-1,1].$$ (Eq 1)

The adjusted RIES, the overall inflammatory effect score (OIES), is calculated by

i) if $\sum_{i=1}^{n}w(x_{pi})<median({\left(\sum_{i=1}^{n}w(x_{pi})\right)}_{p\in P}):$

$${OIES}_{p}=\frac{\sum_{i=1}^{n}w(x_{pi})}{median({\left(\sum_{i=1}^{n}w(x_{pi})\right)}_{p\in P})}\times {RIES}_{p}$$

$$=\frac{\sum_{i=1}^{n}w(x_{pi})}{median({\left(\sum_{i=1}^{n}w(x_{pi})\right)}_{p\in P})}\times \frac{\sum_{i=1}^{n}a(x_{pi})\times w(x_{pi})}{\sum_{i=1}^{n}w(x_{pi})}$$

$$=\frac{\sum_{i=1}^{n}a(x_{pi})\times w(x_{pi})}{median({\left(\sum_{i=1}^{n}w(x_{pi})\right)}_{p\in P})}\in (-1,1)$$

ii) if $\sum_{i=1}^{n}w(x_{pi})\geq median$

$${OIES}_{p}={RIES}_{p}.$$ (Eq 2)

### Global database values

For every food parameter *p*, the global daily consumption is calculated by

$${\bar{I}}_{p}=\frac{1}{l}\sum_{i=1}^{l}I_{p,i},$$ (Eq 3)

where *I*_*p*,*i*_ is the amount (in the same unit) of the daily consumption of the considered food parameter for subject *i* from a global database generated by Shivappa et al. [17].

The according global variability is given by the standard deviation

$${sd}_{p}=\sqrt{\frac{1}{l-1}\sum_{i=1}^{l}{\left(I_{p,i}-{\bar{I}}_{p}\right)}^{2}}.$$ (Eq 4)

### Final calculation steps of the DII

The reported amount of the daily consumption *I*_*p*,*i*_ of a particular food parameter *p* and subject *i* for which the *DII* should be calculated is standardized by

$$Z_{p,i}=\frac{I_{p,i}-{\bar{I}}_{p}}{{sd}_{p}}.$$ (Eq 5)

The *DII* for a particular food parameter *p* and subject *i* results from

$${DII}_{p,i}=(2\times \Phi (Z_{p,i})-1)\times {OIES}_{p}\in [-1,1],$$ (Eq 6)

where Φ is the standard normal distribution function.

Finally, the *DII* for the *i*-th subject is then calculated by

$${DII}_{i}=\sum_{p\in P}{DII}_{p,i}\in [-|P|,|P|],$$ (Eq 7)

completing the original calculation method by Shivappa et al. [17].

### Improvable steps within the original *DII* calculation

In the following, *P* is assumed as the set of food parameters for a set of *n* subjects for which the *DII* should be calculated and $I_{p}\in R_{+}^{n}$ is the vector of the daily consumptions of the parameter *p* ∈ *P*, where the entry *I*_*p*,*i*_ is the daily consumption of the *i*-th subject.

As shown above, the *DII*_*i*_ for the *i*-th subject according to Shivappa et al. is calculated through standardization of the daily consumption *I*_*p*,*i*_ of the subject of the food parameter *p* through first subtracting the global mean ${\bar{I}}_{p}$ from the daily consumption *I*_*p*,*i*_ and then dividing by the global standard deviation *sd*_*p*_ of the considered food parameter. Subsequently, the standardized vector **Z**_*p*_ (Z-scores) is transformed to percentiles of the standard normal distribution function, which are then scaled into [–1,1] and multiplied with the respective effect scores of the food parameter. At last, the scaled and multiplied percentiles are summed across the available food parameter for the *i*-th subject. Here, we focus on the transformation of the daily consumption vector to the percentiles of the standard normal distribution function.

### Insufficient scaling to the entire unit interval

**Fig 1:**
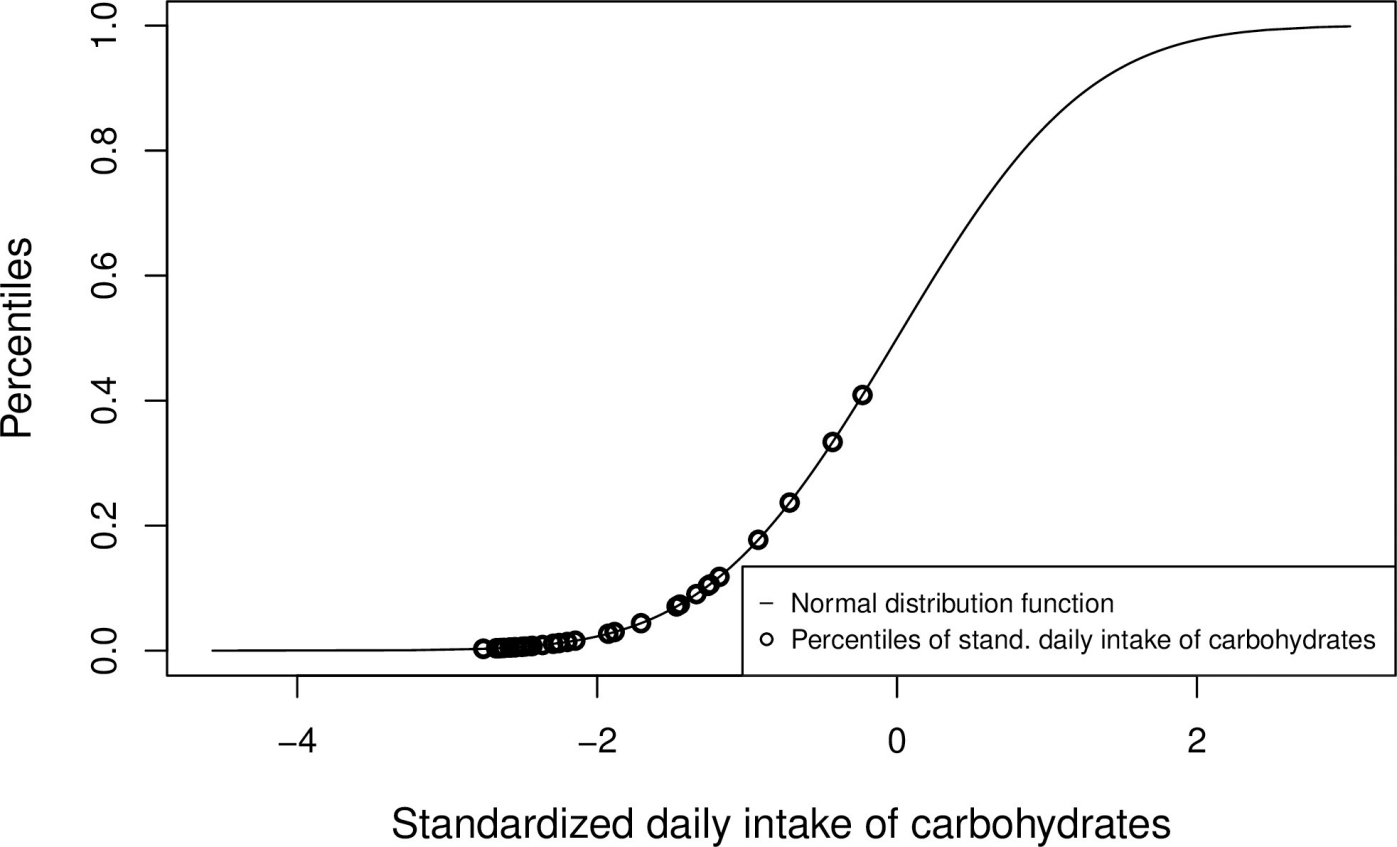
Carbohydrate Z-scores and their corresponding percentiles after transformation using the standard normal distribution function. Due to the usage of the standard normal distribution function by Shivappa et al. [17] to transform daily food parameter Z-scores (here carbohydrates of a simulated dataset of n = 32; Table 1) into percentiles, the resulting percentile scores are only scaled between [0.003, 0.409] and do not distribute across the entire unit interval.

Of note, most of the daily consumptions of a food parameter **I**_*p*_ do not follow a normal distribution by the nature of the data and hence, are not standard normal distributed after standardization with the generated global values. For this reason, the entries of **I**_*p*_ are not transformed through the standard normal distribution function into the entire unit interval but, as expected, in a sub-interval which lies within the entire unit interval. Hence, the unit interval is not fully exhausted through the transformation to percentiles of the standard normal distribution function. To show this effect, we transformed simulated data of the daily intake of a food parameter (Carbohydrates, Table 1) by using the standard distribution function and the resulting percentiles only scaled in the lower part [0.003, 0.409] (Fig 1). All analyses were performed with R software v.4.0.3 (R Foundation for Statistical Computing, Vienna, Austria).

**Table 1:** Simulated data on daily consumption of food parameters and on a pro-inflammatory biomarker used for the Dietary Inflammatory Index (DII) calculation and analyses*.

Subject ID	Carbohydrates (g)	Cholesterol (mg)	Pro-inflammatory biomarker (pg/ml)
1	195.1469	185.1469	35.146869
2	213.3806	203.3806	53.380553
3	172.9380	162.9380	12.937982
4	170.2502	160.2502	10.250178
5	214.2499	204.2499	54.249885
6	167.3584	157.3584	7.358408
7	255.0185	245.0185	95.018535
8	172.0676	162.0676	12.067571
9	203.8999	193.8999	43.899851
10	166.4904	156.4904	6.490351
11	174.5276	164.5276	14.527583
12	221.7369	211.7369	61.736869
13	182.0337	172.0337	22.033748
14	218.6789	208.6789	58.678931
15	165.2294	155.2294	5.229385
16	168.9255	158.9255	8.925473
17	186.3684	176.3684	26.368428
18	224.8152	214.8152	64.815199
19	243.5497	233.5497	83.549694
20	168.8521	158.8521	8.852105
21	196.8460	186.8460	36.846049
22	222.2546	212.2546	62.254598
23	177.6297	167.6297	17.629736
24	170.3257	160.3257	10.325747
25	184.2279	174.2279	24.227861
26	165.7956	155.7956	5.795587
27	235.1728	225.1728	75.172809
28	180.4476	170.4476	20.447607
29	262.9943	252.9943	102.994341
30	174.9587	164.9587	14.958738
31	161.8330	151.8330	1.833000
32	161.8340	151.8340	1.834000

*All variables correlate with each other with a correlation coefficient of r = 1 according to Pearson.

**Fig 2:**
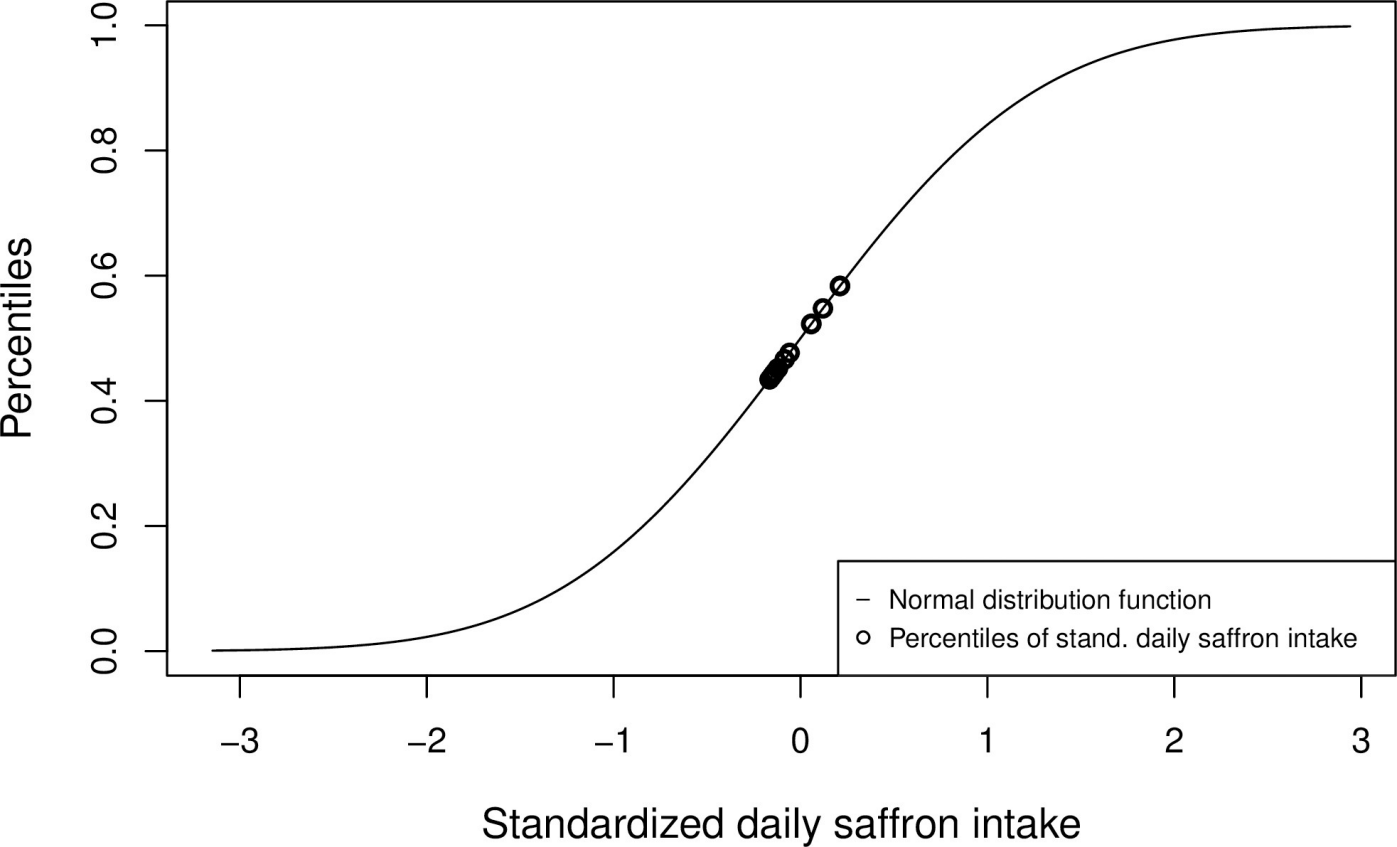
Daily saffron consumption Z-scores and their corresponding percentiles after transformation using the standard normal distribution function. The standardized daily saffron consumption of the subjects (Z-scores of simulated data, n = 13) are scaled into percentiles [0.434, 0.584] when using the method by Shivappa et al. [17]. By using the values ${\bar{I}}_{Saffron}$ = 0.37, *sd*_*Saffron*_ = 1.78 calculated by Shivappa et al. for the standardization, the percentiles cluster in the middle of the standard normal distribution function and do not fill the entire unit interval.

Through the standardization (Eq 5), the daily consumption vector of a specific food parameter is limited through $-\frac{{\bar{I}}_{p}}{{sd}_{p}}$ to the left side and therefore, the percentiles of the standard normal distribution function have a lower limit greater than zero. Furthermore, with increasing right-skewness of the shape of the density function of the vector **I**_*p*_ the percentiles cluster in the lower part [0, 0.5] of the standard normal distribution function. In addition, this effect is amplified if the standard deviation does not fit to the corresponding food parameter. For example, using simulated saffron data, *I*_*Saffron*_ = (0.471, 0.155, 0.109, 0.075, 0.094, 0.266, 0.747, 0.22, 0.153, 0.124, 0.745, 0.583, 0.474), and the values ${\bar{I}}_{Saffron}$ = 0.37, *sd*_*Saffron*_ = 1.78 as calculated by Shivappa et al. for the standardization (see Table 2 in [17]), the resulting percentiles [0.434, 0.584] cluster in the middle part of the standard normal distribution function (Fig 2). The lower limit of percentiles of the standard normal distribution function in this example is $\Phi \left(-\frac{0.37}{1.78}\right)$.

The same effect occurs if the global values are estimated from the compiled data and these data contain outliers. As a consequence, the comparability of the *DIIs* between different studies becomes more difficult because it is assumed that the daily consumption vector is transformed to the entire unit interval but actually the interval is tighter.

### Loss of proportions between subjects

The second improvable aspect is, that through the transformation of the standardized daily consumption vector **I**_*p*_ to percentiles of the standard normal distribution function the proportions between the daily consumptions between the subjects can get lost, even if the daily consumptions **I**_*p*_ of a food parameter would be normal distributed. This can result in unexpected differences between the *DIIs* for subjects with similar nutrition, as proportions should stay equal as well, independent of the amount of food parameter intake (see Table 2 as example). As shown in Table 2, there is a greater difference (around the factor ten) in the *DIIs* according to Shivappa et al. (DII Shivappa) between the subjects with ID = 19 and ID = 27 (ΔID19-27 = 0.020471) than for the subjects with ID = 1 and ID = 17 (Δ1–17 = 0.004511), although the difference of the food parameters between the subjects with ID = 1 and ID = 17 is similar as for the other subjects with ID = 19 and ID = 27. Of note, the within-subject difference between carbohydrates and cholesterol is always the same in this example. Despite a higher intake in subjects ID = 19 and ID = 27, the proportions between the shown subject pairs are expected to be equal. In particular, higher intake amounts can be affected by this effect as the upper scaling interval is often not properly utilized. Like for the previously mentioned issue with different scale intervals of the *DII*, the effect of such differences between the *DII*_*p*,*i*_ for subjects with similar nutrition is amplified with increasing right-skewness of the shape of the density function of **I**_*p*_.

**Table 2:** Differences in the Dietary Inflammatory Index (DII) calculated according to Shivappa et al. [17] or the Scaling-Formula With Outlier Detection (SFOD) method based on similar food consumption data between subject pairs.

Subject ID	Carbohydrates (g)	Cholesterol (mg)	DII Shivappa	DII SFOD
1	195.1469	185.1469	-0.19453557	-0.070663911
17	186.3684	176.3684	-0.19904638	-0.106589436
ΔID1-17	8.778500	8.778500	0.004511	0.035926
19	243.5497	233.5497	-0.12028190	0.127423316
27	235.1728	225.1728	-0.14075287	0.093141146
ΔID19-27	8.376900	8.376900	0.020471	0.034282

### Improvements

#### Refined scaling methods

To avoid these mentioned effects, we suggest a transformation which preserves the proportions between the daily consumptions of the subjects of a specific food parameter and which scales the entries of **I**_*p*_ into the entire unit interval:

$$z_{p,i}=\frac{I_{p,i}-min(I_{p})}{\max\left(I_{p}\right)-min(I_{p})}.$$ (Eq 8)

In the following, this formula will be referenced with *scaling-formula (SF)*.

Indeed, the transformation with the SF depends on the minimum and maximum of **I**_*p*_. Hence, this transformation is more influenced by outliers. To account for outliers the SF can be modified using the interquartile range of the daily consumption vector of a food parameter instead of max(**I**_*p*_) and min(**I**_*p*_). For this, the daily consumption vector of a food parameter should be limited in the following way, where $I_{p_{q}}$ is the *q*-th quartile of **I**_*p*_, $R≔I_{p_{0.75}}-I_{p_{0.25}}$ is the interquartile range, $LL≔I_{p_{0.25}}-1.5\times R$ is the lower limit and $UL≔I_{p_{0.75}}+1.5\times R$ the upper limit:

$${\tilde{I}}_{p,i}=\left\{ \begin{array}{c} LL,I_{p,i}<LL\\ I_{p,i},{LL<I}_{p,i}<UL\\ UL,I_{p,i}>UL \end{array}\right.$$

Hence, the SF is modified to

$$z_{p,i}=\frac{{\tilde{I}}_{p,i}-LL}{UL-LL}$$ (Eq 9)

and is referenced in the following as the *scaling-formula with outlier detection (SFOD)*.

In comparison to the transformation to percentiles by the standard normal distribution function, the proportions between the daily consumptions of the subjects are preserved through the transformation with the SFOD, resulting in more similar *DIIs* between subjects with similar nutrition (see Table 2). As mentioned above, the current calculation method according to Shivappa et al. can result in unequal proportions between subjects with comparable dietary intake. This effect is corrected by the SFOD method (DII SFOD, Table 2), resulting in similar differences (ΔID19-27 = 0.034282, ΔID1-17 = 0.035926).

Moreover, the unit interval is fully utilized by the application of the SFOD method. Furthermore, the SFOD preserves the correlation structure between the *DII* and a pro-inflammatory biomarker (Table 1) with a correlation coefficient of r = 1 according to Pearson in this example, while through the transformation to percentiles of the standard normal distribution function some of the correlation structure gets lost (r = 0.9213171) because of the above-mentioned disadvantages.

### Harmonization

For better comparison of the individual *DIIs* across studies, we suggest to consider the *DII* value for the *i*-th subject relative to the maximum value of the *DII*, which can be taken within a study

$${DII}_{Hi}≔\frac{{DII}_{i}}{\left|P\right|}\in [-1,1].$$ (Eq 10)

## Evaluation of DII calculation methods in the TEENDIAB cohort study

To evaluate the different calculation methods, we used data from the TEENDIAB cohort, a prospective observational cohort study in children and adolescents with at least one first-degree relative with type 1 diabetes. Details of the study have been published previously [40]. Briefly, children were enrolled in the study at the age of 8–12 years and followed until the age of 18 years to investigate the period of puberty and adolescence in the natural course of type 1 diabetes development. The study has been approved by the ethical committee of the Technical University Munich (No. 2149/08) and the Medizinische Hochschule Hannover (No. 5644). Written informed consent was obtained from all participants.

In the current analysis, 193 children with complete data on dietary intake and blood cytokine levels were included. None of the children included was diagnosed with type 1 diabetes. Details of the study cohort are described in Table 3.

**Table 3:** Characteristics of TEENDIAB children/adolescents included in the present analysis.

	Mean ± SD or N (%) (n = 193)
Age (yrs)	10.3 ± 1.2
Females–N (%)	91 (47.2)
BMI-SDS*	0.12 ± 1.22
Weight status†	
Underweight–N (%)	7 (3.6)
Normal weight–N (%)	141 (73.1)
Overweight–N (%)	32 (16.6)
Obese–N (%)	13 (6.7)
Tumor-necrosis factor alpha (pg/ml)	2.76 ± 1.0
Interleukin-6 (pg/ml)	0.54 ± 2.0
Interleukin-10 (pg/ml)	0.48 ± 0.60

*BMI-Standard deviation scores based on age and sex according to WHO reference data [41].

†Weight categories according to BMI-SDS percentiles according to WHO reference data [41].

BMI: Body-mass-index; SDS: Standard deviation score.

### Dietary assessment

Habitual dietary intake was assessed at first study visit using the modified computer-assisted Diet Interview Software for Health Examination Studies Junior (DISHES Junior; Robert Koch Institute, Berlin, Germany). The standardized questionnaire was performed by face-to-face interview with trained staff and collected detailed data on the consumed frequency, type and quantity of foods and beverages of the last four weeks [42]. DIIs according to Shivappa, SF and SFOD methods, as described above, were calculated using total energy intake and the following nutrients/food parameters: alcohol, vitamin B12, vitamin B6, beta-carotene, total carbohydrates, cholesterol, total fat, fiber, folic acid, iron, magnesium, mono-unsaturated fatty acids, niacin, total protein, poly-unsaturated fatty acids, riboflavin, saturated fat, thiamin, vitamin A, vitamin C, vitamin D, vitamin E and zinc. These nutrients/food parameters are the same ones used for the children/adolescent DII [43], with the exception of selenium, which was not assessed in this study.

### Cytokine measurements

To evaluate the different DII calculation methods, we assessed whether the different DIIs were associated with the pro-inflammatory TNF-α and IL-6 and the anti-inflammatory IL-10, which were used by Shivappa et al. for the development of the original DII calculation. Blood samples for analysis of these cytokines were taken at the first study visit and analyzed with Meso Scale Discovery (MSD) electrochemiluminescence assay (Meso Scale Diagnostics, Rockville, MA, USA) at the Institute of Diabetes Research as previously described [44].

### Statistical analyses

Cytokine levels were log-transformed for statistical analyses [44,45]. Linear regression analyses, adjusted for sex and age, were performed to study the associations between DII and cytokine levels.

### Results

**Fig 3:**
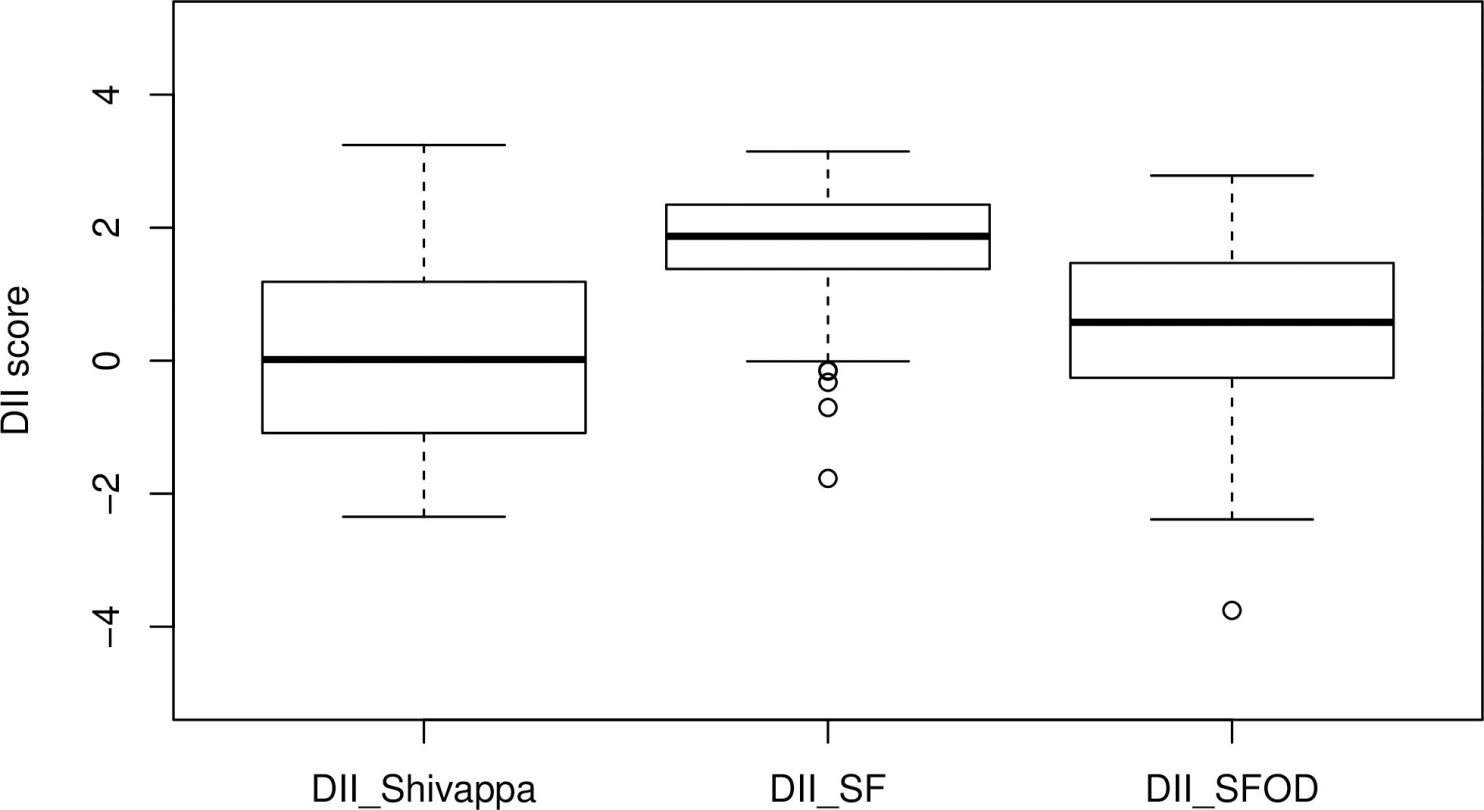
Boxplots of the dietary inflammatory index (DII) scores between the three different calculation methods. Nutritional data from n = 193 subjects participating in the TEENDIAB study were used to calculate the DIIs according to the original method from Shivappa et al. [17] or the revised methods scaling-formula (SF) and scaling-formula with outlier detection (SFOD), respectively.

The distribution of the DII according to the three different calculation methods is shown in Fig 3. The variation of the DII values calculated by the SF and SFOD methods was smaller than the variation of the DII calculated according to Shivappa, while the SF-derived DII showed the smallest variation. Moreover, the median DII score was higher (more pro-inflammatory) when calculated with the revised methods SF and SFOD (Fig 3). While the majority of subjects remained in the same category, i.e., pro- or anti-inflammatory DII, independent of the original or SFOD method, a substantial fraction of 18.1% (n = 35) children changed from a negative DII score according to Shivappa to a positive DII score according to the SFOD method. There was no subject changing from a positive DII score calculated by Shivappa’s method to a negative DII score following the SFOD method. Overall, more children had a proinflammatory DII score according to the SFOD method (n = 132) compared to the method by Shivappa (n = 97).

As shown in Table 4, no significant association was observed between any of the three DII scores (calculated acc. to Shivappa, SF and SFOD, respectively) and TNF-α or IL-6 levels. The DII score, calculated with the SF or SFOD method, was significantly inversely associated with IL-10 levels (Table 4). The same trend was observed when using the DII calculation method proposed by Shivappa et al., although not significant (Table 4).

**Table 4:** Associations between the Dietary Inflammatory Index (DII) calculated according to Shivappa, the Scaling-Formula (SF) and Scaling-Formula With Outlier Detection (SFOD) methods and cytokine levels*.

	Tumor-necrosis factor alpha (pg/ml) (n = 193)		Interleukin-6 (pg/ml) (n = 193)		Interleukin-10 (pg/ml) (n = 193)	
	Coefficients	P-value	Coefficients	P-value	Coefficients	P-value
DII Shivappa	-0.033	0.11	-0.016	0.39	-0.030	0.09
DII SF	-0.049	0.20	-0.030	0.38	-0.073	0.02
DII SFOD	-0.035	0.14	-0.026	0.24	-0.046	0.02

*Data are presented as unstandardized regression coefficients of linear regression analyses adjusted for age and sex in n = 193 subjects of the TEENDIAB study. Cytokine levels were log-transformed.

## Discussion

The application of the DII in a large number of studies in the past years yielded promising results, that this index could be used in the future to estimate the inflammatory potential of someone’s diet and thus, individual risks for several inflammatory-associated diseases [18]. The inventors of the DII already improved the original DII calculation by including energy-adjustment [46] and made it more specific for the application in children [43]. Here, we presented alternative mathematical approaches to further optimize the original DII calculation by Shivappa et al. [17], which also serves as basis for the energy-adjusted and children DII.

With regard to the transformation from standardized Z-scores to percentiles, we demonstrated that using the standard normal distribution function can lead to an incomplete distribution across the whole unit interval and that proportions between the daily food consumptions of different subjects can disappear. Overall, this possibly affects association analyses between the DII and health outcomes in epidemiological and clinical studies. To circumvent these issues, we presented the methods SF/SFOD which capture the lacks of using the standard normal distribution function to scale into the entire unit interval, keep the proportions between subjects and solve the dependency on the global values for the standardization. Simultaneously, a dependency to the used dataset arises and therefore, it would not make sense to calculate the DII with the described SF/SFOD method for a single person. However, this could be easily achieved by the use of the global values developed by Shivappa et al. [17], or alternative (e.g., country-specific) reference values, for the interquartile range (lower and upper limits). An additional benefit of the SFOD method is that any reference data, e.g., age-/sex-/country-specific, can be used. As the DII is usually applied in epidemiological or clinical studies to assess associations between the DII and health outcomes in a defined cohort, the SFOD method should be preferred because of the above described benefits.

While the application of the DII is currently mostly restricted to epidemiological/clinical studies, one aim will be to develop personalized guidelines/recommendations. It remains to be defined what units/cutoffs of the DII will be applied in guidelines/recommendations in the future. There might be more than just the two categories pro- and anti-inflammatory, such as DII scores ranging e.g. from +8 to -8, or the categories high and low pro-inflammatory or anti-inflammatory. Therefore, a more accurate calculation of the individual DII score, as provided by the SFOD method, seems to be more applicable, for example, when monitoring changes of the DII over time.

The comparison of the three different DII calculation methods using data of the TEENDIAB study yielded higher DII scores using the improved SF and SFOD methods, indicating a more pro-inflammatory diet, which is consistent with the previously published observation that dietary patterns in the TEENDIAB cohort were rather “unhealthy” [42]. Thus, participants of the TEENDIAB cohort consumed higher amounts of meat and meat products, sweets, snacks and sweetened beverages and lower amounts of fruits and vegetables than recommended by the optimal mixed diet guidelines [42]. We observed negative associations between the DIIs and the anti-inflammatory cytokine IL-10, as proposed by Shivappa et al. [17]. The observation that the association between DII and IL-10 was stronger when applying the revised DII calculation methods supports the proposed modification of the DII calculation.

Furthermore, no associations were observed between the DIIs and the pro-inflammatory cytokines TNF-α and IL-6 in the TEENDIAB cohort, independent of the applied DII calculation method. Previous studies on the effect of the DII on blood TNF-α/IL-6 levels yielded inconsistent results for children/adolescents; some studies also reported no association [35,38], while another study showed significant associations between the DII and IL-6 [47]. Additional analyses in larger cohorts across all age groups are warranted to validate our findings and show the improvements by the SFOD method.

The evaluation of the three methods in the TEENDIAB cohort has some limitations. First, we used the original DII calculation method instead of the children DII as the global food parameter database for children, that has been used for the calculation method by Shivappa et al., has not been provided with the publication [43]. Still, to calculate the children DII as close as possible, we used the same inflammatory effect scores, which are the same for all DII versions, and the same food parameters that have been suggested for the calculation of the children DII [43], with the exception of selenium intake since it was not assessed in the TEENDIAB cohort. Of note, the original DII has been successfully applied in children/adolescents by the inventors [38,48,49], indicating that the original DII should also be an appropriate measure at young age. Thus, the applied DIIs in the evaluation appear to be valid. Second, C-reactive protein levels, a pro-inflammatory marker used for the validation of the children DII [43], was not available in the TEENDIAB cohort. Therefore, our evaluation is restricted to the provided cytokines.

With the aim to further improve the DII, we focused here on the primary mathematical calculation steps which were made accessible by the inventors in previous publications. The proposed mathematical improvements will affect the calculation of DII at the global level, meaning that they are applicable regardless of age, socio-demographic, or cultural characteristics of the cohort studied. Further improvements may include a weighting algorithm, which bears in mind the influence of the most important food parameters, as the DII according to Shivappa’s calculation does so far not differ between the relevance of the food parameters, i.e., all of them are integrated with the same weight in the DII calculation. For now, we can only speculate if the application of the revised DII calculation would have strongly influenced the findings in the large number of previous publications using the original DII by Shivappa et al., but we think that most of the significant findings might have been stronger/clearer and some findings with borderline significance might have become non-significant. Overall, the revised DII method may provide clearer results in many upcoming analyses.

In summary, we showed a novel approach to improve the DII calculation by Shivappa et al. and provided further steps and suggestions for its optimization. Ultimately, this may increase the potential to identify associations in epidemiological/clinical settings between the DII and inflammatory markers and health outcomes, respectively.
